# Osmotic Demyelination After Rapid Correction of Hyperosmolar Hyperglycemia

**DOI:** 10.7759/cureus.34551

**Published:** 2023-02-02

**Authors:** Evani Jain, Susrutha Kotwal, Jerome Gnanaraj, Waseem Khaliq

**Affiliations:** 1 Internal Medicine, Dayanand Medical College and Hospital, Ludhiana, IND; 2 Internal Medicine, Johns Hopkins Bayview Medical Center, Baltimore, USA; 3 Medicine, Johns Hopkins University School of Medicine, Baltimore, USA

**Keywords:** hyperosmolar hyperglycemic state, rapid hyperglycemia correction, central pontine myelinolysis (cpm), hyperglycemia management, osmotic demyelination

## Abstract

Osmotic demyelination syndrome (ODS) is seen due to an overt rise in serum osmolality, most often during rapid correction of chronic hyponatremia. We present the case of a 52-year-old patient who presented with polydipsia, polyuria, and elevated blood glucose with rapid correction of glucose levels under five hours and developed dysarthria, left-sided neglect, and unresponsiveness to light touch and pain in the left extremities on the second day of hospitalization. MRI revealed restricted diffusion in the central pons, extending into extrapontine areas suggestive of ODS. Our case highlights the importance of cautious correction of serum hyperglycemia and monitoring serum sodium levels in patients with a hyperosmolar hyperglycemic state (HHS).

## Introduction

Osmotic demyelination syndrome (ODS) is a rare, potentially life-threatening neurologic condition that can be triggered by an extreme rise in serum osmolality, most often during rapid correction of chronic hyponatremia [1]. ODS affects myelin and nerve cells in the pons, which can present as muscle weakness, paralysis, speech difficulty, and behavioral changes. Rarely, ODS can be triggered by rapid correction of other osmotic agents [1-5]. We illustrate an unusual case of osmotic demyelination that occurred in association with rapid blood glucose correction in a patient with a hyperosmolar hyperglycemic state (HHS).

## Case presentation

A 52-year-old Caucasian female patient with a significant medical history of insulin-dependent diabetes mellitus, hypertension, hepatitis C virus (HCV), hyperlipidemia, and alcohol and substance abuse disorder on methadone therapy was brought to the emergency department with a four-day history of lethargy, polydipsia, and polyuria in the setting of running out of home insulin and blood pressure medication. At the time of presentation, the patient was afebrile. Her blood glucose level was 1,261 mg/dL, and her blood pressure was 187/102, with low oxygen saturation at room air (80%) requiring supplemental oxygen via nasal cannula. Clinical examination was remarkable for an ill appearance with diffuse abdominal pain and decreased breath sounds bilaterally. Initial blood work revealed the following: sodium of 128 mmol/L (147 mmol/L when corrected for hyperglycemia), potassium of 5.1 mmol/L, chloride of 91 mmol/L, magnesium of 2.8 mg/dL, CO2 of 27 mmol/L, blood urea nitrogen of 21 mmol/L, creatinine of 1.69 mg/dL, albumin of 3.2 g/dL, calculated serum osmolality of 334 mOsm/kg, and hemoglobin A1c > 18%, with pH of 7.37 by venous blood gas and negative serial troponins.

**Figure 1:**
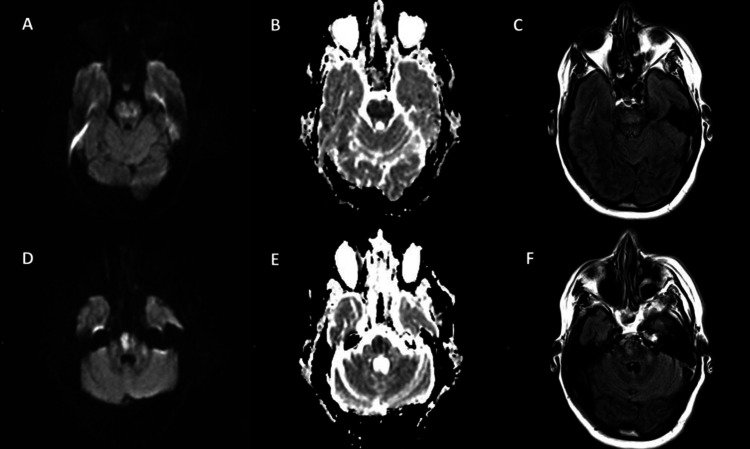
Axial diffusion-weighted imaging (B1000) demonstrating restricted diffusion of the central pons (A and D) with correlate hypointense signal on ADC map (B and E). Correlate T2 FLAIR hyperintense signal (C and F) indicative of cytotoxic edema. ADC: apparent diffusion coefficient, FLAIR: fluid-attenuated inversion recovery

**Figure 2:**
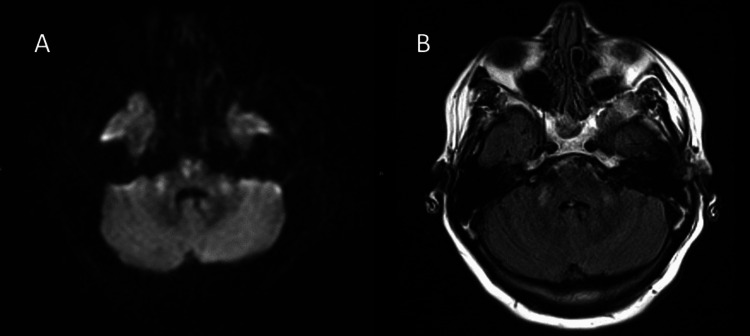
Axial diffusion-weighted imaging (B1000) demonstrating restricted diffusion of the middle cerebellar peduncles (A) correlate T2 FLAIR hyperintense signal (B) indicative of cytotoxic edema. FLAIR: fluid-attenuated inversion recovery

The patient was initially treated with intravenous insulin, intravenous saline, and blood pressure medications for hypertensive urgency and later was admitted to the medicine floor. Initial treatment brought glucose levels down to 608 mg/dL in five hours and to 404 mg/dL in 24 hours. Her Na level was 137 mmol/L ( corrected Na for the degree of hyperglycemia will be 142 mmol/L) in the first 24 hours, and her systolic blood pressure was in the 110s after antihypertensive drugs were administered. Her mental status improved with initial management. However, on day 2 of hospitalization (~40 hours after initial presentation), the patient was found to be lethargic and minimally responsive. The patient’s blood glucose at this time was 233 mg/dL, sodium was 138 mmol/L, and blood pressure was 104/67. On examination, the patient exhibited dysarthria and left-sided neglect and was unresponsive to light touch or pain in the left extremities. Strength was 2/5 in the left upper and lower extremities compared with 4-5/5 on the right. A head CT and CTA ruled out a stroke and acute intracranial abnormality, but MRI revealed restricted diffusion in the central pons suggestive of osmotic demyelination syndrome (Figure 1). The abnormal signal extended into the mid-cerebellar peduncles (Figure 2), and there were further punctate areas of restricted diffusion in the right cerebellar tonsil, right frontal lobe, and right putamen, suggesting extrapontine myelinolysis (EPM). Her hospital course was prolonged by hypertensive urgency and aspiration pneumonia, likely related to weakness, requiring management in the intensive care unit. On discharge, four weeks after her initial presentation, the patient’s dysarthria was much improved, but strength in the left upper and lower extremities remained significantly below baseline.

## Discussion

Our patient presented to the emergency department with lethargy, polydipsia, and polyuria in the setting of noncompliance to antihyperglycemic and antihypertensive medications. After correction of hyperglycemia within the first 40 hours, the patient’s neurologic examination and imaging findings suggest that the patient experienced ODS with both central pontine myelinolysis (CPM) and extrapontine myelinolysis (EPM); however, the underlying cause of the condition in this patient is highly atypical. In over 75% of cases, ODS occurs after rapid correction of hyponatremia, and the mechanism is thought to be related to a relatively hypertonic insult [1,6]. Alcoholism, cirrhosis, malnutrition, and severe burns are some of the other conditions often accompanied by hyponatremia and have been described as risk factors for triggering ODS [1,7]. Our patient’s history of alcoholism and HCV could have impacted her susceptibility. However, the most likely scenario that seemed to have triggered the outcome was HHS. Although rapid correction of hypertensive urgency could confound this picture, due to watershed area infarction, CT and CTA imaging in our patient ruled out the possibility of such an insult or a stroke. Extrapontine myelinolysis associated with hyperosmolar hyperglycemia is uncommon but has been observed in a subset of cases [5].

Current literature reports that some cases of hyperglycemia-associated ODS appear to have been triggered by a rapid rise in serum sodium during correction of HHS [2,3], while in others, including our patient, ODS was associated with a relative hypertonic insult more directly related to the high serum glucose [4-6]. Therefore, cautious correction of hyperglycemia and hydration are the prime variables in the prevention of ODS. Treatment is mainly supportive. There have been some experimental therapies that have been tried with mixed results. All of them are based on immune modulation using plasmapheresis, intravenous immunoglobulin (IVIg), and corticosteroids or a combination of these [8-10].

## Conclusions

In conclusion, osmotic demyelination syndrome can be a rare but severe consequence of overly rapid correction of serum osmolality and glucose in HHS, even in the absence of severe hyponatremia or rapid change in sodium levels. While lowering glucose levels and hydration remain the mainstay of treatment in HHS, it is of prime importance to closely monitor and correct any rapid electrolyte fluctuations. Cautious correction of serum hyperglycemia and careful attention to corrected serum sodium may aid with the prevention of this neurologic complication, and brain MRI should be considered to aid diagnosis in suspected cases.
